# Modified Grape Seeds: A Promising Alternative for Nitrate Removal from Water

**DOI:** 10.3390/ma14174791

**Published:** 2021-08-24

**Authors:** Marija Stjepanović, Natalija Velić, Mirna Habuda-Stanić

**Affiliations:** Faculty of Food Technology Osijek, Josip Juraj Strossmayer University of Osijek, Franje Kuhača 18, HR-31000 Osijek, Croatia; natalija.velic@ptfos.hr (N.V.); mirna.habuda-stanic@ptfos.hr (M.H.-S.)

**Keywords:** nitrate removal, adsorption, adsorbent, grape seeds, column study

## Abstract

The aim of this work was to investigate grape seeds as a potential adsorbent for nitrate removal from water. Grape seeds were modified by quaternization and the applicability of the modified grape seeds (MGS) was evaluated in batch adsorption experiments. Fixed bed adsorption and regeneration studies were carried out to determine the regeneration capacity of MGS. The maximum adsorption capacity of 25.626 mg g^−1^ at native pH (6.3) for nitrate removal by MSG was comparable to that of the commercial anion exchange resin Relite A490 under similar conditions. The percent removal of nitrate from model nitrate solution was 86.47% and 93.25% for MGS, and Relite A490, respectively, and in synthetic wastewater 57.54% and 78.37%. Analysis of the batch adsorption data using isotherm models revealed that the Freundlich model provided a better fit to the data obtained than the Langmuir model, indicating multilayer adsorption. In kinetic terms, the results showed that the adsorption followed the pseudo-first order model. By investigating the adsorption mechanism, the results suggest that the intraparticle diffusion model was not the only process controlling the adsorption of nitrate on MGS. In column experiments (adsorption/desorption studies), three adsorption cycles were tested with minimal decrease in adsorption capacities, implying that this alternative adsorbent can be successfully regenerated and reused.

## 1. Introduction

The daily increase of nitrate in surface and groundwater is a cause for concern in many parts of the world because of the negative impact on water bodies and human health [1]. The highest nitrate concentrations have been found in the areas of intensive agricultural production [2]. The natural nitrate concentration in groundwater depends on the type of soil and geology. Soil bacteria convert nitrogen to nitrate, and due to its high leachability, nitrate can easily migrate with water through the soil profile and eventually reach groundwater [3]. Consumption of water with excessive nitrate concentrations can cause health problems, such as methemoglobinemia in infants, diabetes, vomiting, diarrhea, central nervous system birth defects, cancer, and other diseases [4,5,6]. Considering that, numerous health problems are related to excessive nitrate concentration in drinking water, and therefore the World Health Organization (WHO) and the U.S. Environmental Protection Agency (U.S. EPA) have established a limit of 10 mg L^−1^ NO_3_-N in drinking water. Efficient nitrate removal from water is an urgent need to meet these regulations.

To date, physical, chemical, and biological methods have been developed for nitrate removal from water. Physico-chemical methods (reverse osmosis, adsorption, ion exchange, electrodialysis and catalytic reactions [7,8,9]) are effective in removing nitrate ions, but their application is limited due to high operating costs. Biological denitrification is an adequate choice in wastewater treatment, but is relatively slow and requires the disposal of activated sludge. Of the above methods, adsorption is still the preferred method and is mostly used on an industrial scale [10]. Activated carbon is one of the most studied adsorbents used for water treatment, but often the main drawback of commercially available activated carbons is their price and problems with regeneration, i.e., regeneration is not straightforward and may result in reduced adsorption capacity. An alternative to this could be low-cost, but often equally efficient adsorbents produced from various lignocellulosic waste materials from agri-food industry [11,12]. Large quantities of lignocellulosic by-products and wastes are generated daily in the agri-food industry. In the context of the circular economy model, these lignocellulosic materials can be considered as a valuable resource that can be used for many purposes, from the production of bioactive compounds to energy production and use as adsorbents to remove pollutants from wastewater. The removal of pollutants (adsorbates) from water using lignocellulosic materials as adsorbents is achieved by their interaction with functional groups (e.g., hydroxyl and carboxyl), which are abundant in these materials and also provide a good basis for the preparation of anion exchange resins [13]. Various treatment methods for lignocellulosic waste materials have been investigated to increase the number and availability of their functional groups. Such modification methods include the use of organic or inorganic acids, oxidizing agents, alkali or salt solutions, and many more [14]. The modification using ethylenediamine and triethylaminein in the presence of *N*,*N*-dimethylformamide (ETM method) is a commonly used crosslinking method that is effective for the preparation of strongly basic anion exchangers [15]. Nitrate ions are highly soluble and stable in water, so adsorption occurs mainly by electrostatic attraction or ion exchange [16]. Some studies show that a material modified by quaternary amine groups with longer alkyl groups acquires a stronger hydrophobic property, making it preferable to anions with lower hydration energy, such as nitrate ions, and repulsive to anions with higher hydration energy, such as sulfate ions [17]. Considering that lignocellulosic polymers constitute a large part of grape seeds, they can be considered as promising candidates for the modification and preparation of an efficient adsorbent for nitrate removal. Therefore, the main objective of this work was to modify the grape seeds by quaternization using ETM method and evaluate the obtained modified grape seeds as a potential adsorbent for nitrate removal. Furthermore, the adsorption performance of modified grape seeds (MGS) for nitrate removal was comparatively investigated with a commercial adsorption/ion exchange resin Relite A490. The effect of different process parameters was investigated in batch adsorption experiments. In addition, the MGS were also tested in column experiments and the regeneration capacity was investigated to determine the practical applicability of the MGS.

## 2. Materials and Methods

### 2.1. Materials

All chemicals used in this study were of analytical grade. Ethylenediamine and epichlorohydrin (ECH) were obtained from Sigma Aldrich (St. Louis, MO, USA), triethylamine from Fisher Scientific (Leicestershire, UK) and *N*,*N*-dimethylformamide (DMF) from GramMol (Zagreb, Croatia). Potassium nitrate (Merck, Darmstadt, Germany) was used to prepare the nitrate solutions. A stock solution of 1000 mg L^−1^ (as N-NO_3_^−^) was prepared by dissolving 7.218 g KNO_3_ in 1 L demineralized water. The experimental solutions were prepared by diluting the stock solution to the desired concentration (10–300 mg L^−1^). 

Synthetic wastewater was prepared by dissolving nutrients and minerals in demineralized water according to OECD guidelines 302 [18]. The composition of the synthetic wastewater was as follows: peptone (160 mg L^−1^), meat extract (110 mg L^−1^), urea (30 mg L^−1^), K_2_HPO_4_ (28 mg L^−1^), NaCl (7 mg L^−1^), CaCl_2_·2H_2_O (4 mg L^−1^), MgSO_4_·7H_2_O (2 mg L^−1^). For the preparation of synthetic wastewater with the addition of nitrate, the required amount of nitrate was added to the prepared synthetic wastewater up to a final nitrate mass concentration of *γ*_nitrate_ = 30 mg L^−1^. The pH of the synthetic wastewater with the addition of model nitrate solution was pH = 7.22 (Seven Easy, Mettler Toledo, Greifensee, Switzerland).

A commercial adsorption/ion exchange material (Relite A490, Resindion S.r.l. Binasco, Italy) was used for comparison purposes. Table 1 summarizes the properties of the Relite A490 anion exchange resin.

### 2.2. Preparation of MGS

Grape seeds were obtained from the Faculty of Agrobiotechnical Sciences Osijek (Osijek, Croatia). The material was ground using a laboratory knife mill with a 1 mm sieve (MF10 basic, IKA Labortechnik, Staufen, Germany) and then sieved using a vibrating sieve shaker (AS 200 Digit, Retsch GmbH, Haan, Germany). The sieve analysis showed that the highest percentage of the total mass of sieved samples were particles ranging from 318 to 380 µm, so this particle range was chosen for the modification process.

The chemical modification method of GS was previously described by Stjepanović et al. [19], namely 2 g GS was reacted with 16 mL DMF and 13 mL ECH at 70 °C for 45 min. Then, 2.5 mL of ethylenediamine was added and stirred for another 45 min at 80 °C. Finally, 13 mL of triethylamine was added and the mixture was stirred at 80 °C for 120 min. The modified GS (MGS) was washed thoroughly with Milli-Q water and dried for 24 h at 100 °C. 

### 2.3. Characterization of MGS

Elemental composition (C, H, and N) was performed using a Perkin Elmer CHNS/O analyzer (II series, Waltham, MA, USA). The surface morphology of the unmodified and modified GS was examined using a field emission scanning electron microscope (FE SEM, JOEL, JSM-7000 F, Akishima, Tokyo, Japan). Fourier transform infrared spectroscopy (FT-IR) was performed using a FTIR spectrometer (Cary 630, Agilent Technologies, Santa Clara, CA, USA).

### 2.4. Batch Experiments

A conventional batch adsorption method was used to investigate the adsorption of nitrate on MGS. All experiments were carried out in duplicates and were found to be reproducible. A detailed description is given below, where *γ*_0_ is the initial concentration of nitrate, *γ*_adsorbent_ is the concentration of adsorbent used, *t* is the contact time, *T* is the temperature, *v* is the speed of the thermostatic shaker, and *V* is the volume of the aqueous phase (nitrate solution).

(a) Effect of initial MGS or Relite A490 concentration: *γ*_0_ = 30 mg L^−1^, *γ*_adsorbent_ = 1–10 g L^−1^, *V* = 50 mL, pH = native (6.3), *T* = 25 °C, *t* = 120 min, *v* = 130 rpm.
(b) Effect of contact time: *γ*_0_ = 30 mg L^−1^, *γ*_adsorbent_ = 4 g L^−1^, *V* = 50 mL, pH = native (6.3), *T* = 25 °C, *t* = 2–360 min, *v* = 130 rpm.
(c) Effect of initial nitrate concentration: *γ*_0_ = 10–300 mg L^−1^, *γ*_adsorbent_ = 4 g L^−1^, *V* = 50 mL, pH = native (6.3), *T* = 25 °C, *t* = 120 min, *v* = 130 rpm.
(d) Effect of initial pH: *γ*_0_ = 30 mg L^−1^, *γ*_adsorbent_ = 4 g L^−1^, *V* = 50 mL, pH = 2, 4, 6, 7, 8, 10, *T* = 25 °C, *t* = 120 min, *v* = 130 rpm.
(e) Effect of contact time (synthetic wastewater): *γ*_0_ = 30 mg L^−1^, *γ*_adsorbent_ = 4 g L^−1^, *V* = 50 mL, pH = native (7.2), *T* = 25 °C, *t* = 2–360 min, *v* = 130 rpm.

The experiments were carried out in a thermostatic shaker (Poly-test 20, Bioblock Scientific, Illkirch, France). During the adsorption experiments, samples were taken at predetermined time intervals, filtered, and the residual nitrate concentrations were determined spectrophotometrically using a UV/Vis spectrophotometer (Specord 200, Analytic Jena, Jena, Germany) at 324 nm.

The percentage of nitrate removal *R* (%) was calculated as follows:

(1) $$R=\frac{\gamma_{0}-\gamma }{\gamma_{0}}\cdot 100\%$$

where *γ*_0_ and *γ* (mg/L) are the initial nitrate concentration and nitrate concentration after predetermined contact time, respectively.

The uptake of nitrate was quantified based on the following mass balance equation:

(2) $$q_{e}=\frac{\left(\gamma_{0}-\gamma_{e}\right)}{m}\cdot V$$

where *q*_e_ is the amount of nitrate adsorbed (mg g^−1^), *γ*_0_ and *γ*_e_ (mg L^−1^) are the initial nitrate concentration and concentration at equilibrium, *V* is the solution volume (L), and *m* is the mass of MGS or Relite A490. The effect of temperature was studied at 25, 35 and 45 °C. The isotherm and kinetic models were used to fit the data. 

### 2.5. Column Experiments

To investigate the feasibility of using MGS in practical applications, fixed-bed experiments were performed using a glass column of 13 mm internal diameter and 220 mm total length packed with 1 g of MGS. 2 L of a 30 mg L^−1^ nitrate solution was pumped from top to bottom through the bed using a peristaltic pump (Masterflex L/S, Cole-Palmer Instrument Company, Vernon Hills, IL, USA) at a controlled constant flow rate of 10 mL min^−1^. Experiments were performed at 25 °C and natural solution pH. 100 mL of samples were collected at the bottom of the column and the nitrate concentration was determined. After the adsorption process, the spent MGS or Relite A490 were regenerated in situ with 200 mL of 0.1 M NaCl and 500 mL of demineralized water at a flow rate of 10 mL min^−1^. The saturation capacity was calculated as follows:

(3) $$q_{s}=\frac{\gamma_{0}V_{0}-\sum \gamma_{n}V_{n}}{m}$$

where *γ*_0_ is the initial nitrate concentration (mg L^−1^), *V*_0_ is the initial volume of nitrate solution (L), *γ*_0_ is the nitrate concentration in fraction *n* (mg L^−1^), *V*_*n*_ is the volume of fraction *n* (L) and *m* is the mass of MGS (g).

## 3. Results and Discussion

### 3.1. Characterization of MGS

A novel adsorbent for the removal of nitrate was prepared by modification of GS using ETM method. 2 g of GS yielded 10 g of the modified adsorbent (MGS). The characterization of GS and MGS is given below.

The elemental analysis of the unmodified and modified GS can be seen in Table 2. The nitrogen content of MGS is higher than that of GS and increased from 2.03 to 9.83%, which is due to the introduction of ammonium groups during the modification process. This is in agreement with other results reported elsewhere [3,20].

**Figure 1:**
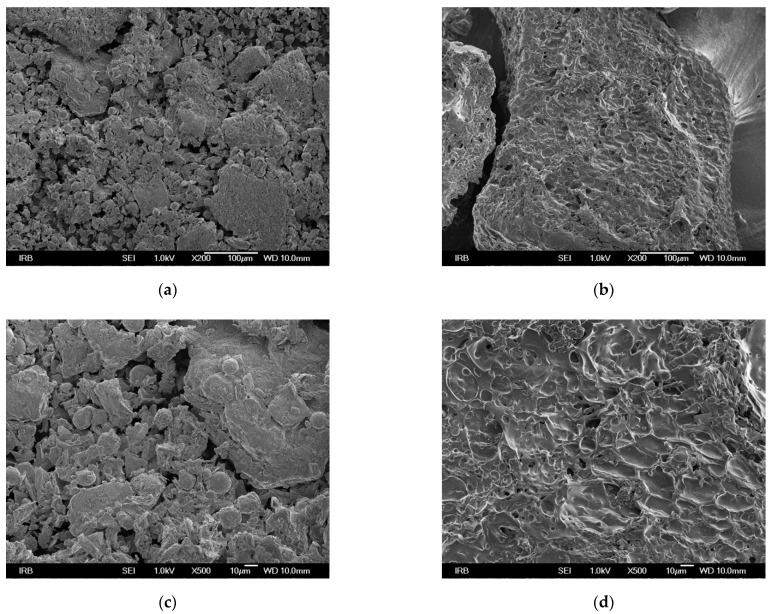

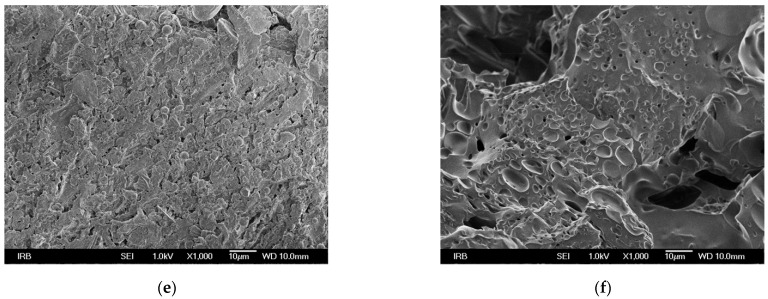
FESEM micrographs of grape seeds at different magnifications (**a**) 200, (**c**) 500, (**e**) 1000, and of Relite A490 at (**b**) 200, (**d**) 500, (**f**) 1000.

FESEM imaging was used to investigate the morphological characteristics of the surface. The microscopic images are shown in Figure 1. They show that the surface of MGS is more heterogeneous and rougher, and has more cavities compared to GS.

**Figure 2:**
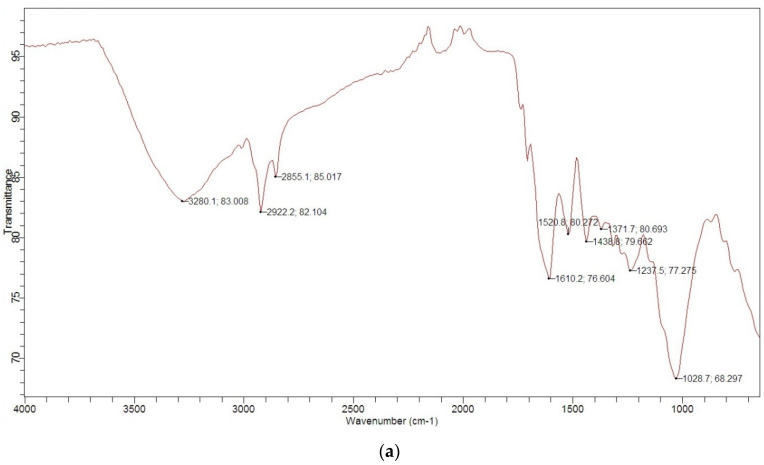

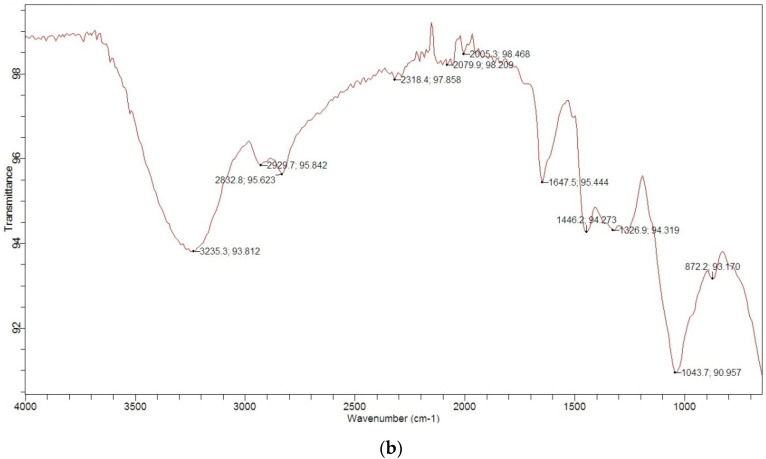
FTIR spectra of (**a**) GS and (**b**) MGS.

FTIR spectra of GS and MGS were recorded to investigate the presence of surface functional groups (Figure 2). The FTIR spectra of GS showed the presence of -OH groups at 3280 cm^−1^, where the very broad bands can be attributed to the OH stretching vibrations, probably due to inter- and intramolecular hydrogen bonding of the polymers (lignin and cellulose). The peaks at 2922 cm^−1^ and 2855 cm^−1^ could be attributed to the C-H stretching vibrations of -CH_2_ and CH_3_ groups. After modification of GS, the vibrational frequency of -CH_2_ groups was shifted to 2832 cm^−1^. The band at 1446 cm^−1^ in MGS is associated with quaternary ammonium groups [21]. A broad band at 1043 cm^−1^ corresponds to the vibration of quaternary ammonium salt [22]. The occurrence of the peak at 872 cm^−1^ in MGS can be attributed to glycoside bonds deformed by vibration and OH bending [23]. Similar results were obtained for quaternary amino anion exchangers from wheat residues [24] and giant reed used for phosphate removal [15].

### 3.2. Applications of MGS for the Removal of Nitrate from Water

Numerous factors, such as contact time, initial adsorbate concentration, adsorbent concentration, pH and temperature, can affect the adsorption process differently. 

**Figure 3:**
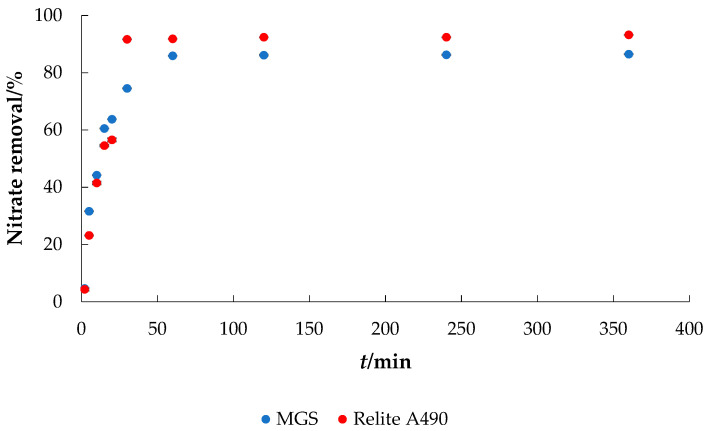
The effect of contact time on the adsorption of nitrate to MGS and Relite A490 (*γ*_0_ = 30 mg L^−1^, *γ*_adsorbent_ = 4 g L^−1^, pH = 6.3, *T* = 25 °C, *v* = 130 rpm).

The effect of contact time on nitrate removal from water (Figure 3) was studied from 2–1440 min. As can be seen from Figure 3, the nitrate removal efficiency increased with increasing contact time. During the first 30 min, over 75% of nitrate ions were removed with MGS and about 92% with Relite A490 anion exchange resin. The adsorption capacities were up to 6.5 and 6.8 mg g^−1^ for MGS and Relite A490, respectively. Equilibrium was reached within 60 min. However, in all further experiments, a contact time of 120 min was chosen to ensure equilibrium was reached. Similar results were obtained with quaternary starch derivatives, where the results showed an initial rapid increase in adsorption from 35 to 86.5% [1]. Compared to the nitrate selective anion exchanger Relite A490, MGS showed slightly lower removal efficiency. However, it can still be considered a very good alternative to commercial anion exchangers. The main mechanism responsible for the adsorption of nitrate on MSG was probably ion exchange, which is also supported by the study of Keränen et al. [13], who determined the chlorine concentration after adsorption and concluded that the increased Cl^−^ concentration came from the ETM-modified pine sawdust.

**Figure 4:**
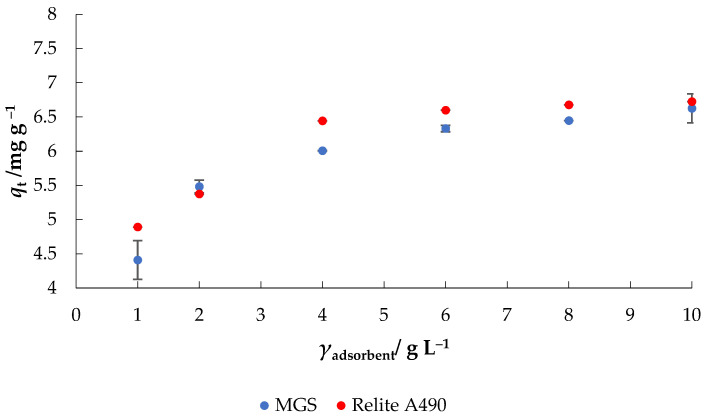
The effect of adsorbent concentration on the adsorption of nitrate to MGS and Relite A490 (*γ*_0_ = 30 mg L^−1^, *t* = 120 min, pH = 6.3, *T* = 25 °C, *v* = 130 rpm).

The adsorption of nitrate anion increased with the increase of adsorbent concentration and reached a maximum value at 10 g L^−1^ MGS (Figure 4). The increase in percentage nitrate removal was due to the large number of active sites available for adsorption. The highest removal efficiency of 90% was obtained at an adsorbent concentration of 10 g L^−1^ with MGS, and 94% with Relite A490 at the same ion exchange concentration. From Figure 4, it can be seen that the increase of adsorbent concentrations over 4 g L^−1^ for both tested adsorbents had no significant effect on the removal efficiency of nitrate ions, so the concentration of 4 g L^−1^ was chosen for further experiments. Similar trends were reported by Kalaruban et al. [25,26] who tested Dowex 21K XLT and Dowex 21K XLT ion exchange resins with Fe and amine grafted agricultural wastes, and Hekmatzadeh et al. [27] by testing commercial IND NSSR resin against nitrate removal: the increase in the nitrate removal efficiency is due to a larger exchangeable area for nitrate adsorption.

**Figure 5:**
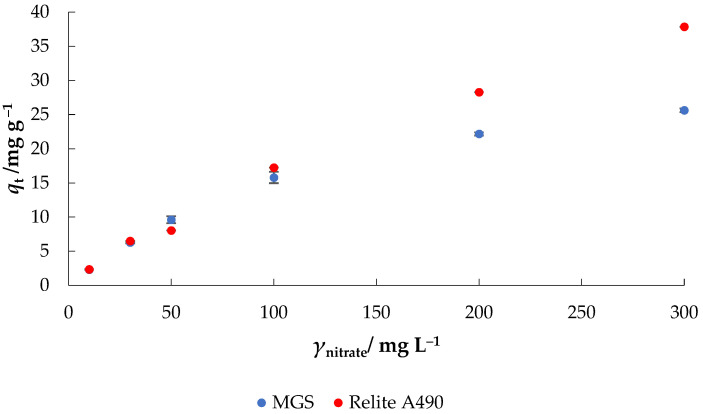
The effect of initial nitrate concentration on the adsorption of nitrate to MGS and Relite A490 (*γ*_0_ = 10–300 mg L^−1^, *γ*_adsorbent_ = 4 g L^−1^, *t* = 120 min, pH = 6.3, *T* = 25 °C, *v* = 130 rpm).

The effect of initial nitrate concentration was investigated at different concentrations (10–300 mg L^−1^) as shown in Figure 5. The adsorption capacity increased with the increase of initial nitrate concentration from 2.28 to 25.62 mg g^−1^ and from 2.35 to 37.91 mg g^−1^ using MGS and Relite A490, respectively. The results are in accordance with the previously published results confirming that adsorptive removal of nitrate is concentration dependent [21]. The mass transfer resistance between the liquid and solid phases can be overcome by a higher driving force, which is achieved at higher nitrate concentrations. On the other hand, the decrease in the percentage removal of nitrate is attributed to the exhaustion of adsorption sites of MGS and Relite A490. There are a number of researches reporting the same trend. For example, Sowmya and Meenakshi [28] reported that the adsorption capacity of quaternized chitosan-melamine-glutaraldehyde resin increased significantly with increasing nitrate concentration; the maximum adsorption capacity was 97.5 mg g^−1^.

**Figure 6:**
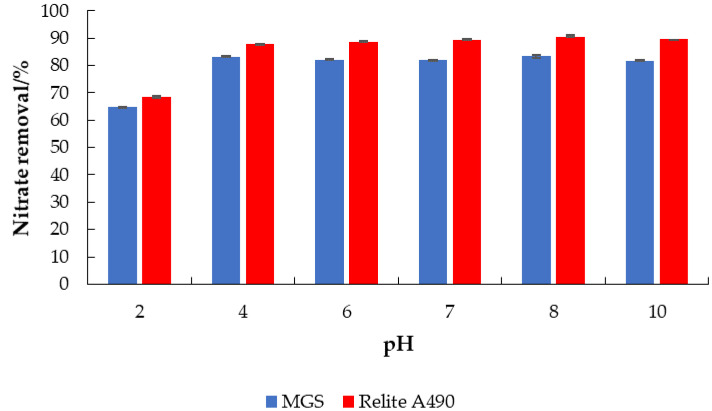
The effect of pH on the adsorption of nitrate to MGS and Relite A490 (*γ*_0_ = 30 mg L^−1^, *γ*_adsorbent_ = 4 g L^−1^, *t* = 120 min, pH = 6.3, *T* = 25 °C, *v* = 130 rpm).

One of the major factors in the adsorption of nitrate from water is the pH of the solution. The pH of a solution controls the electrostatic interactions between the adsorbate and the adsorbent [10]. Figure 6 shows the effect of initial pH on adsorptive nitrate removal from a model nitrate solution using MGS and Relite A490. In the pH range of 4 to 10, the maximum percent nitrate removal was achieved. At pH 2, lower nitrate removal was observed, which can be explained by the competition of chloride ions with nitrate for adsorption sites (Cl- forms HCl solution at pH adjustment) [29]. In the work of Sowmya and Meenakshi [30], nitrate removal was studied over the pH range of 3 to 9 using quaternized chitosan beads. Again, the percentage nitrate removal was lower at pH 2. Shojaipour et al. [31] studied nitrate removal with functionalized hydrogel bioadsorbents (GT) over a broad pH range (3–10), and revealed best nitrate adsorption at neutral pH, and low nitrate removal at acidic pH due to hydration of the hydroxyl group of the GT. 

### 3.3. Adsorption of Nitrate onto MGS from Real Wastewater

**Figure 7:**
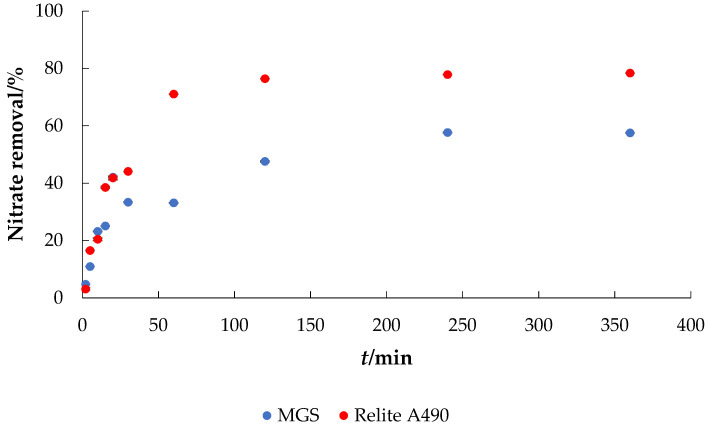
Adsorption of nitrate to MGS and Relite A490 from synthetic wastewater (*γ*_0_ = 30 mg L^−1^, *γ*_adsorbent_ = 4 g L^−1^, *t* = 120 min, pH = 7.2, *T* = 25 °C, *v* = 130 rpm).

Model solutions are usually used to study the applicability of an adsorbent, especially when low-cost (unconventional) adsorbents are involved. However, the composition of real wastewater is much more complex and has many contaminants that can affect the adsorption process. To further explore the possible use of MGS as adsorbent, the adsorption of nitrate on MGS from synthetic wastewater with the addition of nitrate was investigated and the results are shown in Figure 7.

The percent removal of nitrate from the synthetic wastewater after 360 min was 57.53% for MGS and 78.37% for Relite A490 (compared to Figure 3, the removal efficiency from the model nitrate solution was 86.5% for MGS and 93.3% for Relite A490). The decrease in removal efficiency from the synthetic wastewater was expected and can probably be attributed to the competing ions present. In particular, sulfate occupies most of the binding sites on adsorbent/anion exchange resins at concentrations commonly present in many water supply sources, which may further reduce the volume of an influent that can be treated before regeneration of adsorbent/anion exchange resins is required [32]. Banu and Meenakshi [33] reported that the sorption capacities of quaternized melamine-formaldehyde (MFQ) resin for nitrate were changed by Cl^−^ and much more by SO_4_^2−^. The SO_4_^2−^ ion has a higher anionic charge than nitrate, i.e., a multivalent anion with greater charge density was adsorbed faster than a monovalent anion.

### 3.4. Adsorption Kinetics

The kinetics (Table 3) describes the rate of adsorbate uptake at the solid-liquid interface and can be used to design appropriate adsorption technologies. Table 4 summarizes the characteristic parameters and correlation coefficients obtained from the intercept and slope of the nonlinear plots. The correlation coefficients (*R*^2^) and the agreement between the experimental data (*q*_e exp_) and the calculated data (*q*_e cal_) indicate that the adsorption of nitrate fits best with the pseudo-first model (Lagergren model), which is in accordance with other research [33,34]. The applicability of the pseudo-first order model assumes the formation of a monomolecular nitrate layer on the surface of MGS and Relite A490 [35]. The Elovich model takes into account that the solid surface is energetically heterogeneous and sorption occurs through a chemical reaction [36].

**Figure 8:**
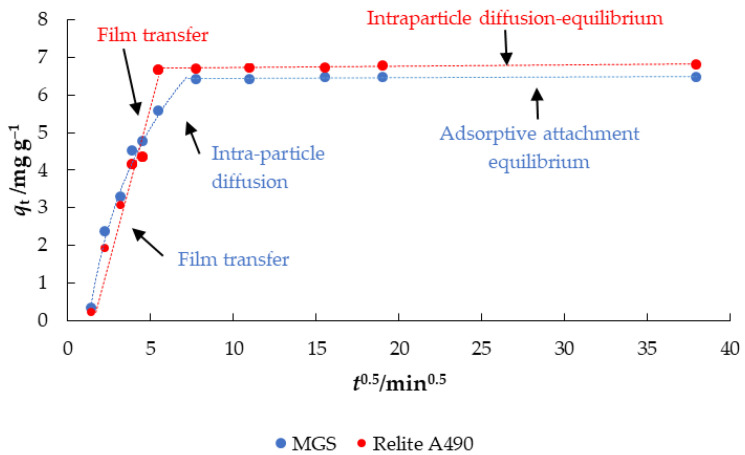
Intraparticle diffusion plots for nitrate removal using MGS and Relite A490 (*γ*_0_ = 30 mg L^−1^, *γ*_adsorbent_ = 4 g L^−1^, *t* = 2–1440 min, pH = 6.3, *T* = 25 °C, *v* = 130 rpm).

Diffusion kinetic models describe mass transport from adsorbates to adsorbents, explaining different transport pathways and suggesting rate-controlling steps. The plot of the intraparticle diffusion model shows multilinearity, indicating the occurrence of two steps during the adsorption process (Figure 8).

According to Weber [39], the first step here is film transport (film diffusion), in which the adsorbate is transported from the liquid bulk to the outer surface of the adsorbent, the second step is intraparticle diffusion, in which the adsorbate diffuses from the outside of the adsorbent into the pores, and the third step the adsorptive attachment equilibrium phase, in which the adsorbate binds to the active sites of the adsorbent for MGS, while both film transport and intraparticle diffusion equilibrium can be observed for Relite A490. The intraparticle diffusion rate model of Weber and Morris [40] is presented below:

(4) $$q_{t}=k_{i}\sqrt{t}+C$$

where *q*_t_ (mg g^−1^) is the amount of adsorbate adsorbed at time *t* (min), *k*_i_ (mg g^−1^ min^0.5^) is the intra-particle diffusion model constant and *C* (mg g^−1^) is a constant associated with the thickness of the boundary layer (the higher the C, the thicker boundary layer).

Table 5 shows the modeled diffusion parameters (*k*_i_ and *C*) for nitrate removal using MGS and Relite A490. The values for *k*_i1_ were 1.6787 and 1.4737 mg g^−1^ min^−0.5^ for MGS and Relite A490, respectively. The second phase for MGS was intraparticle diffusion with k_i2_ 0.6744, while for Relite A490 it was intraparticle-equilibrium phase with k_i2_ 0.0033 mg g^−1^ min^−0.5^, while *C*_2_ was 6.6936. The third phase for MGS indicates equilibrium because of the low adsorbate concentration in the solution, which may explain the lower *k*_i3_ (0.002 mg g^−1^ min^−0.5^), and thicker boundary layer (*C*_3_ = 6.4193).

### 3.5. Adsorption Isotherms 

**Figure 9:**
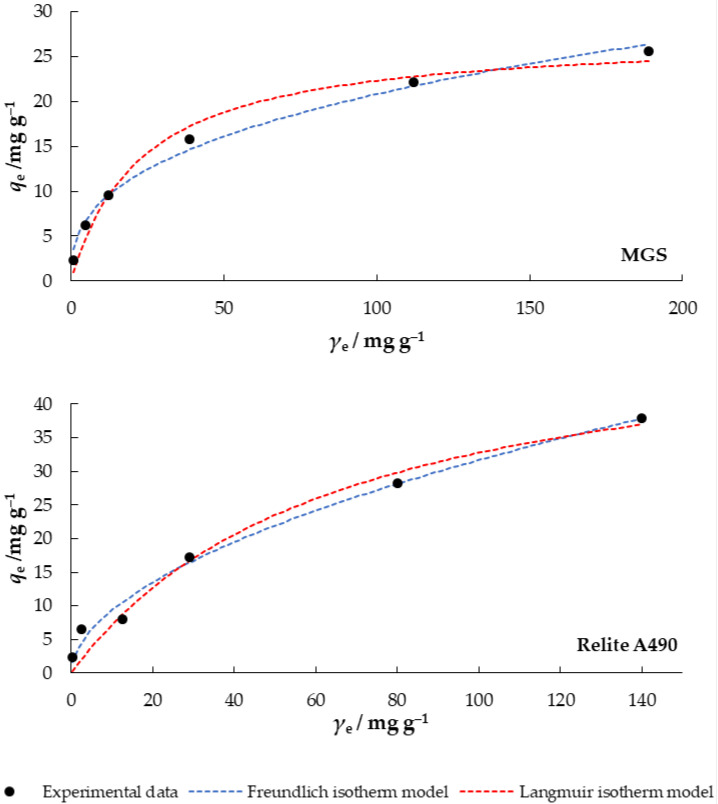
Langmuir and Freundlich isotherms of nitrate adsorption on MGS and Relite A490 (*γ*_0_ = 30 mg L^−1^, *γ*_adsorbent_ = 4 g L^−1^, *t* = 120 min, pH = 6.3, *T* = 25 °C, *v* = 130 rpm).

Adsorption isotherms provide information about the distribution between liquid and solid phases in equilibrium and give insight into the maximum adsorption capacity of an adsorbent for adsorbate [41]. For this purpose, the Langmuir and Freundlich adsorption isotherms are usually used. Figure 9, therefore, shows the adsorption isotherms for nitrate on MGS and Relite A490.

The Langmuir adsorption isotherm model assumes the formation of a monolayer on the adsorbent surface, a uniform adsorption energy along the entire surface, and no interaction between the adsorbed molecules. The Langmuir equation can be expressed as follows [42]:

(5) $$q_{e}=\frac{q_{m}\cdot K_{L}\;\cdot \gamma_{e}}{1+K_{L}\cdot \gamma_{e}}$$

where *γ*_e_ (mg L^−1^) is the nitrate concentration at equilibrium, *q*_e_ (mg g^−1^) is the amount of nitrate adsorbed per unit mass of adsorbent, *q*_m_ (mg g^−1^) is the maximum amount of nitrate adsorbed and *K_L_* (L mg^−1^) is the Langmuir constant. The dimensionless constant *R_L_* (equilibrium parameter) indicates the type of isotherm to be favorable (0 < *R_L_* < 1), unfavorable (*R_L_* > 1), linear (*R_L_* = 1) or irreversible (*R_L_* = 0). It can be calculated as follows [42]:

(6) $$R_{L}=\frac{1}{1+K_{L}\cdot \gamma_{o}}$$

where *γ*_0_ (mg L^−1^) is the highest initial concentration of nitrate. The *R*_L_ values presented in Table 6 are 0.074 and 0.137 (for MGS and Relite A490), which indicates that adsorption of nitrate on MGS and Relite A490 under the applied experimental conditions was a favorable process. 

To describe adsorption in a multilayer, on heterogeneous surfaces, where interactions between adsorbate molecules occur, the Freundlich adsorption isotherm is used [42].

(7) $$q_{e}=K_{f}{\gamma_{e}}^{1/n}$$

where *q*_e_ (mg g^−1^) is the adsorbed amount of nitrate ions at equilibrium, *γ*_e_ (mg L^−1^) is the concentration of nitrate in solution at equilibrium, *K_f_* is the constant indicating adsorption capacity of adsorbent and *n* indicates the intensity of adsorption. When *n* < 1 adsorption is a chemical process, *n* = 1 adsorption is linear, and *n* > 1 indicates a physical process that is favorable. The values of the constant *n* in this study were 2.688 and 1.885 for MGS and Relite A490, respectively, which indicates a physical process that is favorable. 

By analyzing the Langmuir and Freundlich isotherm plots for nitrate adsorption on MGS shown in Figure 9 and the *R*^2^ values for both models given in Table 6, it seems that the Freundlich model slightly better describes the experimentally obtained data. Similar results were obtained by Ogata et al. [43] with soybean treated with calcium chloride, hydrochloric acid, and calcination (at 600 °C). Results obtained by Karachalios and Wazne [22] on quaternized pine bark to remove nitrate from water, showed that Langmuir model (*R*^2^ = 0.999) provided slightly higher correlation coefficients than Freundlich model (*R*^2^ = 0.955), which indicated that the adsorption process was monolayer on a surface with finite number of binding sites.

### 3.6. Breakthrough and Desorption Studies

**Figure 10:**
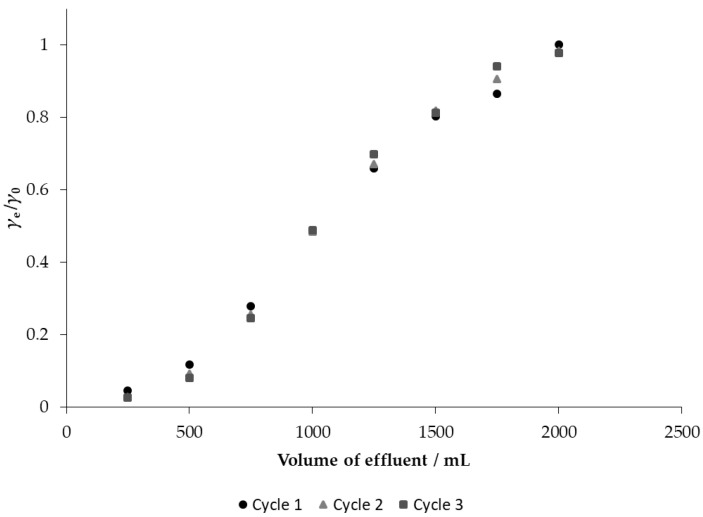
Breakthrough curves for the adsorption of nitrate from model nitrate solution on MGS (bed volume 4 mL, *γ*_0_ = 30 mg L^−1^).

The applicability of MGS on nitrate removal was investigated in fixed bed column experiments. The breakthrough curves obtained are shown in Figure 10. One of the desirable characteristics of a good adsorbent is to be reusable over several adsorption/desorption cycles after regeneration [44], so three adsorption and desorption cycles were performed in this study using MGS as adsorbent.

Approximately 97% nitrate removal from the influent was observed in the model nitrate solution. After the second and third cycles, only a slight decrease in adsorption capacity can be observed, from which it can be concluded that the adsorbent is stable and reusable. The saturation capacity after the first cycle was 27.90 mg g^−1^, while after the second and third cycles it was 27.79 mg g^−1^ and 27.67 mg g^−1^, respectively, which is an indicator that the adsorbent could perform well after the regeneration process. Similar results were observed by Stjepanović et al. [19] who investigated modified brewers’ spent grain against nitrate removal and Karachalios and Wazne [22] who investigated quaternized pine bark against nitrate removal.

## 4. Conclusions

This study investigated the applicability of grape seeds for adsorptive removal of nitrate from water. The nitrate adsorption efficiency was investigated in a model nitrate solution and synthetic wastewater. Adsorption of nitrate was rapid and reached equilibrium within 60 min. The adsorption was efficient in a pH range of 4 to 10. The Freundlich isotherm model slightly better described the experimentally obtained data, suggesting multilayer (physical) adsorption. Kinetically, the pseudo-first order model provided a slightly better fit for the systems studied. The highest uptakes of 25.626 mg g^−1^ (MGS) and 37.910 mg g^−1^ for MGS and Relite A490, respectively, were obtained. The kinetics data were also fitted with the intraparticle diffusion kinetics model, and the obtained results indicated the occurrence of at least two steps during the adsorption process. The adsorption of nitrate from synthetic wastewater showed that the percent removal of nitrate is somewhat lower than in model nitrate solution probably due to other ions who compete for adsorption sites. The column studies showed that MGS could be successfully regenerated. 

Modified grape seeds showed promising results in nitrate removal from model solutions and synthetic wastewater and the results highlight the potential of using waste materials in the removal of pollutants from water from the viewpoint of clean, sustainable and practical water treatment.

## Figures and Tables

**Table 1:** Characteristics of the commercial strongly basic selective resin Relite A490 used in this study.

Typical Characteristics	
Matrix	Porous copolymer styrene-DVB
Functional group	Quaternary ammonium group
Color and physical form	Light yellowish opaque beads
Particle size range	0.3–1.18 mm
Effective size	0.4 mm
Uniformity coefficient	1.7 max
Ionic form	Cl^−^
Total exchange capacity	0.9 min eq/L
Water retention	46–54%
Chemical stability	Stable in the whole pH range
Thermal stability	100 °C max (Cl^−^ form)

**Table 2:** Elemental composition of GS and MGS.

Parameter % Mass	GS	MGS
C	48.61	45.10
H	6.11	9.01
N	2.03	9.83

**Table 3:** Kinetic models examined in this research.

Kinetic Model	Formula	Reference
Pseudo-first order	$\ln\left(q_{e\;}-q_{t}\right)=lnq_{e}-k_{1}t$.	[37]
	where *q*_e_ is the amount of dye adsorbed at equilibrium, *q*_t_ (mg g^−1^) is the amount of nitrate adsorbed at time *t* (min) and *k*_1_ (min^−1^) is the pseudo-first-order rate constant.	
Pseudo-second order	$\frac{1}{q_{t}}-\frac{1}{q_{e}}=\frac{1}{k_{2}\cdot q_{e}^{2}\cdot t}$.	[38]
	where *k*_2_ (g mg^−1^ min^−1^) is the pseudo-second-order rate constant	
Elovich	$q_{t}=\frac{1}{\beta }\cdot \ln\left(\alpha \beta \right)+\frac{1}{\beta }\ln\left(t\right)$	[36]
	where *α* (mg g^−1^ min^−1^) is Elovich constant indicative of the initial adsorption rate, *β* (mg g^−1^) is Elovich constant indicative of the desorption constant	

**Table 4:** Adsorption kinetic parameters for nitrate onto MGS and Relite A490.

Kinetic model	Parameter	Adsorbent	
		MGS	Relite A490
	*q*_e exp_ (mg g^−1^)	6.488	6.805
Pseudo-first order	*q*_e cal_ (mg g^−1^)	6.451	6.847
	*k*_1_ (min^−1^)	0.073	0.062
	*R* ^2^	0.987	0.974
Pseudo-second order	*q*_e cal_ (mg g^−1^)	6.863	7.304
	*k*_1_ (g mg^−1^ min^−1^)	0.016	0.012
	*R* ^2^	0.969	0.935
Elovich	*α* (mg g^−1^ min^−1^)	4.618	3.115
	*β* (mg g^−1^)	1.114	0.990
	*R* ^2^	0.763	0.745

**Table 5:** Parameters of the Weber and Morris intraparticle diffusion model for the removal of nitrate by MGS and Relite A490 (*γ*_0_ = 30 mg L^−1^, *γ*_adsorbent_ = 4 g L^−1^, *t* = 2–1440 min, pH = 6.3, *T* = 25 °C, *v* = 130 rpm, * mg g^−1^ min^−0.5^).

Intraparticle Diffusion Model									
Parameter									
Adsorbent	*k*_i1_ *	*C*_1_	*R*_1_^2^	*k*_i2_ *	*C*_2_	*R*_2_^2^	*k*_i3_ *	*C*_3_	*R*_3_^2^
**MGS**	1.6787	<0	0.9431	0.6744	1.855	0.9758	2·10^−3^	6.4193	0.6496
**Relite A490**	1.4737	<0	0.9776	3.3·10^−3^	6.6936	0.7043	N/A	N/A	N/A

**Table 6:** Isotherm parameters for the removal of nitrate by MGS at 25 °C.

Isotherm Model	MGS	Relite A490
*q*_m exp_./mg g^−1^	25.626	37.910
**Langmuir**		
*q*_m cal_./mg g^−1^	27.468	54.183
*K_L_*/L mg^−1^	0.043	0.015
*R*_*L*_	0.074	0.137
*R* ^2^	0.981	0.971
*MSE*	1.353	4.679
*RMSE*	1.163	2.163
**Freundlich**		
*K_f_*/(mg g^−1^ (L/mg)^1/*n*^)	3.756	2.752
*n*	2.688	1.885
*R* ^2^	0.991	0.988
*MSE*	0.661	1.919
*RMSE*	0.813	1.385

## Data Availability

Data Sharing is not applicable.
